# Protein-bound Uremic Toxin Lowering Effects of Sevelamer in Hyperphosphatemic Hemodialysis Patients: A Multicenter Randomized Controlled Trial

**DOI:** 10.1016/j.xkme.2026.101527

**Published:** 2026-09-11

**Authors:** Napas Srifa, Jeerath Phannajit, Patchara Tanateerapong, Kamonrat Chongthanakorn, Sipanan Thamratnopkoon, Wannasit Wathanavasin, Supat Vanichakarn, Sookruethai Lekhyananda, Yingyos Avihingsanon, Kearkiat Praditpornsilpa, Somchai Eiam-Ong, Paweena Susantitaphong

**Affiliations:** 1 Division of Nephrology, Department of Medicine, Chulalongkorn University, Bangkok, Thailand; 2 Center of Excellence for Metabolic Bone Disease in CKD Patients, Chulalongkorn University, Bangkok, Thailand; 3 Division of Clinical Epidemiology, Department of Medicine, Chulalongkorn University, Bangkok, Thailand; 4 Nephrology Unit, Department of Medicine, Charoenkrung Pracharak Hospital, Bangkok, Thailand; 5 The Kidney Foundation of Thailand, Bangkok, Thailand

**Keywords:** Arterial stiffness, calcium carbonate, chronic kidney disease, hemodialysis, hyperphosphatemia, p-cresyl sulfate, phosphate binders, protein-bound uremic toxins, residual kidney function, sevelamer

## Abstract

**Rationale & Objective:**

p-Cresyl sulfate (pCS) is an important protein-bound uremic toxin associated with high cardiovascular mortality in patients receiving long-term hemodialysis (HD). Data regarding pCS lowering and the pleiotropic effects of sevelamer are limited in patients receiving long-term HD.

**Study Design:**

Open-label, multicenter randomized controlled trial.

**Setting & Participants:**

Patients with hyperphosphatemia receiving long-term HD.

**Exposures:**

Patients were randomly assigned to receive either sevelamer or calcium carbonate as phosphate binders for 24 weeks.

**Outcomes:**

The primary outcome was the between-group difference in pCS change over 24 weeks. Secondary outcomes included changes in high-sensitivity C-reactive protein levels, intact parathyroid hormone levels, cardio-ankle vascular index, and adverse event rates.

**Analytical Approach:**

Mean differences between groups were analyzed using a linear mixed model. Multivariate logistic regression was used to determine factors associated with decreased pCS levels.

**Results:**

A total of 128 patients were enrolled, 4 dropped out, and 124 were analyzed. Residual kidney function (RKF) ≥100 mL/day was present in 47.7%. Both groups achieved significant phosphate reduction. Mean changes in pCS did not differ significantly between groups: 0.70 mg/L (95% CI, –1.04 to 2.44) in the sevelamer group versus –0.09 mg/L (95% CI, –1.70 to 1.51) in the calcium carbonate group (*P* = 0.07). However, sevelamer was associated with a smaller increase in cardio-ankle vascular index than calcium carbonate (adjusted mean change difference –0.63 [95% CI, –1.22 to –0.04]; *P* = 0.04). Preserved RKF and high-dose phosphate binders were independently associated with decreased pCS levels.

**Limitations:**

Change in pCS is only a surrogate marker for cardiovascular events. The study may have been underpowered, or the sevelamer dose and treatment duration may have been insufficient.

**Conclusions:**

pCS levels were not significantly changed in both groups. However, the increase in cardio-ankle vascular index was smaller with sevelamer than with calcium carbonate, independent of pCS change. Further trials with larger samples and longer treatment durations are warranted.

## Plain-Language Summary

People on long-term hemodialysis can build up harmful waste products in the blood, including p-cresyl sulfate, which has been linked to heart and blood vessel problems. We studied whether sevelamer, a common phosphate binder, could lower this toxin and provide added benefits compared with calcium carbonate. In this multicenter randomized trial, adults receiving long-term hemodialysis with high phosphate levels received one of these treatments for 24 weeks. Both treatments lowered phosphate levels, but neither clearly lowered p-cresyl sulfate levels more than the other. However, sevelamer was linked to less worsening in a measure of arterial stiffness. Preserved residual kidney function and higher phosphate binder doses were also linked to lower toxin levels. These findings may help inform future dialysis care and research.

## Graphical Abstract

**Figure:**
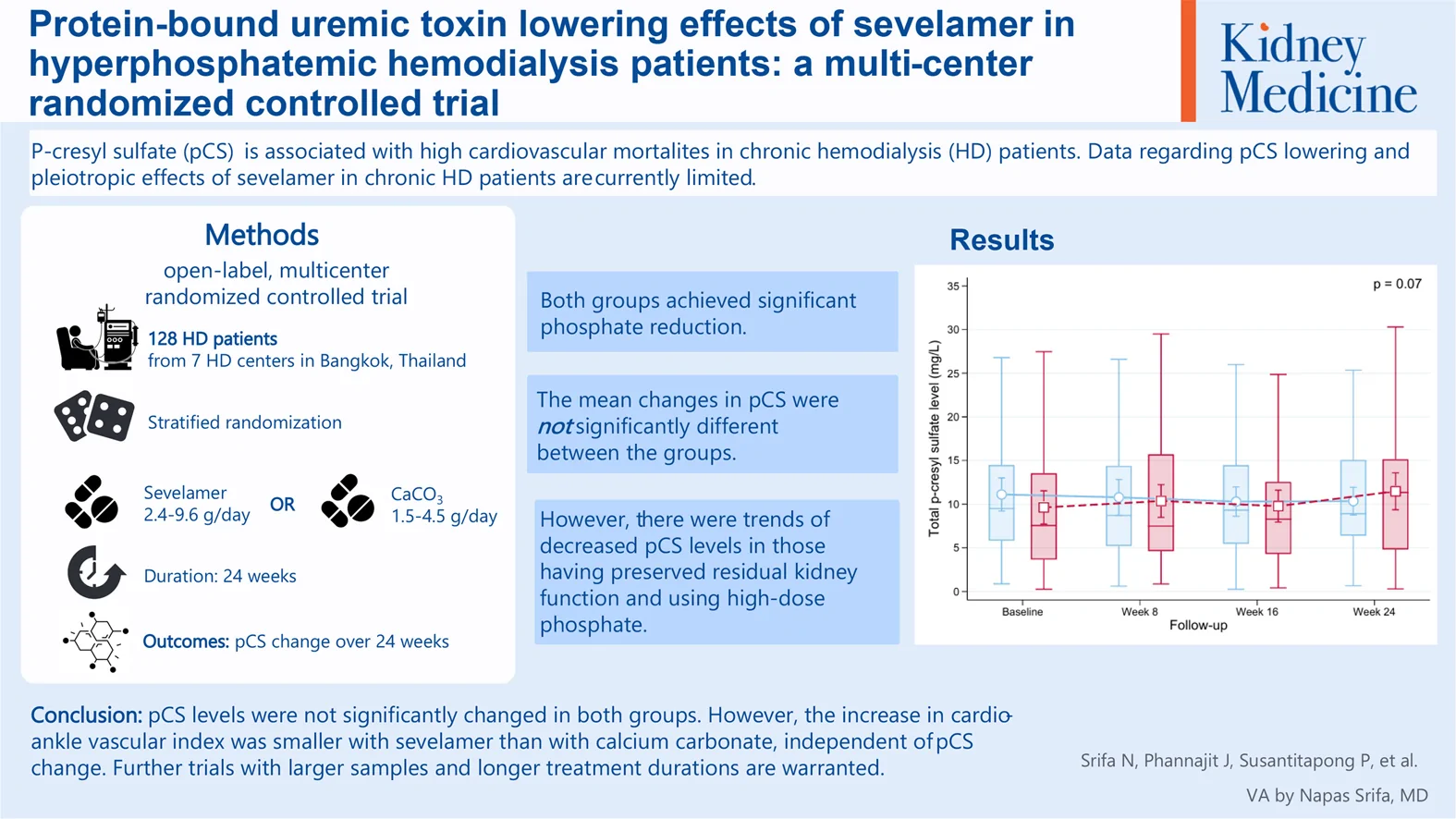

Cardiovascular diseases are leading complications in patients with kidney failure receiving replacement therapy (KFRT) receiving long-term hemodialysis (HD).^1^^,^^2^ These complications arise from traditional risk factors such as advanced age, diabetes, hypertension, and dyslipidemia, as well as dialysis-related factors, including vascular access complications, electrolyte imbalances, anemia, inflammation, mineral and bone disorders (MBD), and solute retention. Uremic toxins are categorized as small molecules, middle molecules, and protein-bound uremic toxins (PBUTs).^3^

p-Cresyl sulfate (pCS) and indoxyl sulfate are the 2 most significant PBUTs generated by colonic bacteria fermentation. Their accumulation is driven by high protein intake, alterations in gut microbiota, and increased intestinal permeability because of uremic states.^4^ Although pCS is normally eliminated via renal tubular active transport, this pathway fails as kidney function declines and progressive loss of residual kidney function (RKF) in patients receiving long-term HD.^5^ Paradoxically, the 1.0-1.2 g/kg/day protein intake recommended by the Kidney Disease Outcomes Quality Initiative (KDOQI) to maintain nutritional status inadvertently increases the substrate available for toxin production.^6^ This creates a high-risk state in which toxin generation is maximal, whereas both natural and HD modalities produce minimal clearance because of the high albumin binding (>90%) of these toxins.^7^, ^8^, ^9^ Consequently, serum levels can reach 50-fold higher than in healthy individuals.^3^^,^^7^ Preclinical data indicate that pCS increases vascular permeability and exerts direct cardiotoxicity through inflammatory and fibrotic pathways, a pathophysiology shared by indoxyl sulfate.^10^, ^11^, ^12^, ^13^, ^14^, ^15^

Current research focuses on decreasing PBUT generation, increasing gut absorbent capacity, and preserving RKF.^16^, ^17^, ^18^, ^19^, ^20^, ^21^, ^22^ Sevelamer, an anion exchange resin used as a phosphate binder, has demonstrated pleiotropic potential by adsorbing pCS in the gut. Although randomized clinical trials (RCTs) in predialysis CKD have shown conflicting results, a recent RCT by Takkavatakarn et al^17^ demonstrated that sevelamer was more effective than calcium carbonate in lowering pCS levels in predialysis CKD when combined with protein restriction.^23^^,^^24^ Furthermore, a cohort study in HD patients observed significant pCS reduction after three months of sevelamer (800 mg thrice daily for 3 months).^18^

Whether the pCS-lowering effect of sevelamer translates into a mortality benefit in patients receiving HD remains an important and unresolved question. Prior large epidemiologic analyses of United States kidney failure registry data have not demonstrated a survival benefit of sevelamer over calcium-based phosphate binders.^25^^,^^26^ However, these studies are often confounded by indication and did not assess PBUTs levels as an intermediary mechanism. Therefore, a controlled trial specifically designed to evaluate pCS reduction as a primary endpoint is warranted to determine whether sevelamer’s mechanistic advantage in reducing PBUTs is achievable in the HD setting. This RCT is the first to compare the pCS-lowering and pleiotropic effects of sevelamer with calcium carbonate when used as phosphate binders in patients receiving long-term hemodialysis and addresses the key mechanistic and clinical knowledge gaps that prior epidemiologic studies were not designed to resolve.

## Materials and Methods

### Study design

We conducted a prospective multicenter, open-label RCT across 7 HD centers in Bangkok from July 1, 2023, to June 30, 2024.

### Setting and participants

We included patients with KFRT aged 18 years or older who had been undergoing maintenance high-flux HD for more than 3 months and had shown adherence to HD treatment but still had persistent hyperphosphatemia (≥5.5 mg/dL) in 2 consecutive blood tests or hyperphosphatemia after 2 weeks of phosphate binder discontinuation.

The exclusion criteria were pregnancy, patients with active malignancies or expected life expectancy less than 24 weeks, or patients who had planned kidney transplantation in the next 24 weeks. Furthermore, those who had serum calcium >10.0 mg/dL without calcium supplement, uncontrolled intact parathyroid hormone >1,000 IU/mL during screening period, recent stroke within 6 months, or a history of amputation or revascularization because of severe peripheral arterial disease were also excluded because of contraindications to calcium supplementation.

This study was approved by the Institutional Review Board, Faculty of Medicine, Chulalongkorn University (IRB No. 0056/66; Approval No. 0644/2023), and conducted in accordance with the Declaration of Helsinki and the International Conference on Harmonisation Good Clinical Practice guidelines. The study was registered with the Thai Clinical Trials Registry (identifier: TCTR20230530004).

### Randomization

The randomization sequence was generated using the Sealed Envelope blocked randomization list generator (Sealed Envelope Ltd. 2024) with variable block sizes of 2, 4, and 6 stratified by HD frequency (twice or thrice weekly). Randomization was implemented in REDCap, and patients were assigned to either the sevelamer or calcium group. Laboratory technicians were blinded to treatment assignment.

### Intervention and dosing regimen

During the 2-week run-in period, all phosphate binders were discontinued, and a low-phosphate diet (≤800 mg/day) was advised. Patients with persistent hyperphosphatemia (serum phosphate ≥5.5 mg/dL) were then randomized to sevelamer carbonate (Kavolred® 800 mg) or calcium carbonate (Chalkcap® 1,000 mg containing 400 mg elemental calcium). Doses were titrated to maintain serum phosphate levels at 3.5-5.5 mg/dL per protocol (Appendix S1), with dialysate calcium adjusted to maintain serum calcium levels at 8.3-10.5 mg/dL. Withdrawal from the study was considered for drug intolerance, persistent hypophosphatemia, or hypercalcemia in 2 consecutive tests. Treatment allocation was maintained throughout the study; no crossover between treatment arms was permitted.

Concomitant pharmacological and nonpharmacological treatments, such as antihypertensive agents, intravenous iron, and erythropoietin-stimulating agents, were adjusted according to patient conditions. Active vitamin D analogs and calcimimetic agents were not adjusted during the study. All patients underwent 4-hour high-flux HD twice or thrice weekly, targeting a minimum single-pool Kt/V of 2.1 and 1.2, respectively. Reused dialyzers followed standard guidelines.

### Data collection and outcome measures

Demographic data, comorbid conditions, dialysis vintage, RKF, and medication history were collected. At baseline and 24 weeks, blood biochemistry (pre- and postdialysis urea, creatinine levels, electrolyte levels, iPTH levels, liver function tests, lipid profiles, hemoglobin A1c (HbA1c) levels, and complete blood count), cardio-ankle vascular index, and ankle-brachial index were measured. Vascular assessments were performed before midweek HD using a VaSera VS-2000 device (Fukuda-Denshi, Tokyo, Japan). During follow-up (baseline and weeks 8, 16, and 24), serum pCS, calcium, phosphate, albumin, and high-sensitivity C-reactive protein (hs-CRP) levels were measured alongside clinical evaluation and pill count. At week 4, phosphate, calcium, and albumin levels were additionally assessed for dose titration. Prespecified adverse events (constipation, nausea, diarrhea) were recorded by patient report. Total pCS was quantified using high-performance liquid chromatography (HPLC; intra-assay coefficient of variation 1.65%), iPTH using chemiluminescence immunoassay, and hs-CRP by latex agglutination.

The primary outcome was the between-group difference in pCS change over 24 weeks. Secondary outcomes included changes in hs-CRP levels, iPTH levels, cardio-ankle vascular index, and adverse event rates. Adherence below 80% of prescribed doses was defined as nonadherence.

### Statistical analysis

Sample size estimation was based on the results of previous study of Takkavatakarn et al^17^, by using mean total pCS change difference between sevelamer and calcium group as the main variable and by assuming 80% power and 2-sided alpha of 5%. By allowing 20% of dropped out patients during the study, the final sample size would be 70 patients in each arm.

Demographic data were presented as mean ± standard deviation or median with interquartile range for continuous data, depending on distribution, and as ratios and percentages for categorical data. Patient characteristics were compared using chi-square, unpaired *t* test, or Mann–Whitney U test as appropriate. A linear mixed-effects model for repeated measures was used to assess treatment effects among patients receiving sevelamer or calcium carbonate, including data from baseline, 8th, 16th, and 24th weeks. A random intercept model was used with time coded as a continuous factor, assuming that individual variation was primarily at baseline. A random slope was excluded because of model convergence constraints. P-interactions for each subgroup were also analyzed using this model. Analysis of covariance was used to compare end-of-study values of end-of-study biochemical outcomes and cardio-ankle vascular index change adjusting for respective baseline values. Subgroup analyses for primary and secondary outcomes were conducted based on RKF ≥100 mL/day or less, high or low dosages of phosphate binders (calcium carbonate > 3 g/day or sevelamer > 4.8 g/day), mean phosphate level >5.5 mg/dL or less, and normalized protein catabolic rate (nPCR) > 1.2 g/kg/day or less. Skewed data for pCS, hs-CRP and iPTH levels were log-transformed for analysis. As data were log-transformed, results are expressed as adjusted geometric means with 95% confidence intervals (CIs) derived via back-transformation (exponentiation) of the least-squares means and its confidence limits from the linear mixed model. A two-sided *P* value of less than 0.05 was considered statistically significant.

In addition, univariable logistic regression was used to identify variables, including type of phosphate binders, HD frequency, RKF, nPCR, mean phosphate level, and phosphate binder dosages (high dosages were predefined as sevelamer more than 4.8 g/day or calcium carbonate more than 3 g/day), associated with increased pCS levels. The increased pCS levels were determined by positive average pCS slope over 24 weeks. Then variables with *P* value less than 0.25 were selected for multivariable logistic regression using backward elimination to demonstrate significant factors attributed to increased total pCS. Sensitivity analyses were also performed by excluding noncompliant patients. Dropped-out patients were excluded before the analysis as per-protocol basis. STATA SE version 18.0 (Stata Corp, College Station, TX, USA) was used for all analyses in this study.

## Results

### Study population

Among 575 HD patients, we identified 180 patients who were eligible for run-in by discontinuing phosphate binders. After a run-in period, 128 patients with persistent hyperphosphatemia were randomized to receive either sevelamer carbonate or calcium carbonate as phosphate binders. One patient from calcium carbonate group declined to participate after randomization because of personal concern. Three patients died after randomization to sevelamer group, including 1 patient who died from myocardial infarction after 2 weeks, 1 patient who died from stroke after 9 weeks, and another from blunt abdominal trauma after 8 weeks follow-up. Therefore, only 124 remaining patients, including 60 patients in the calcium group and 64 patients in the sevelamer group, completed follow-up and were included in the analysis (Fig 1).

**Figure 1:**
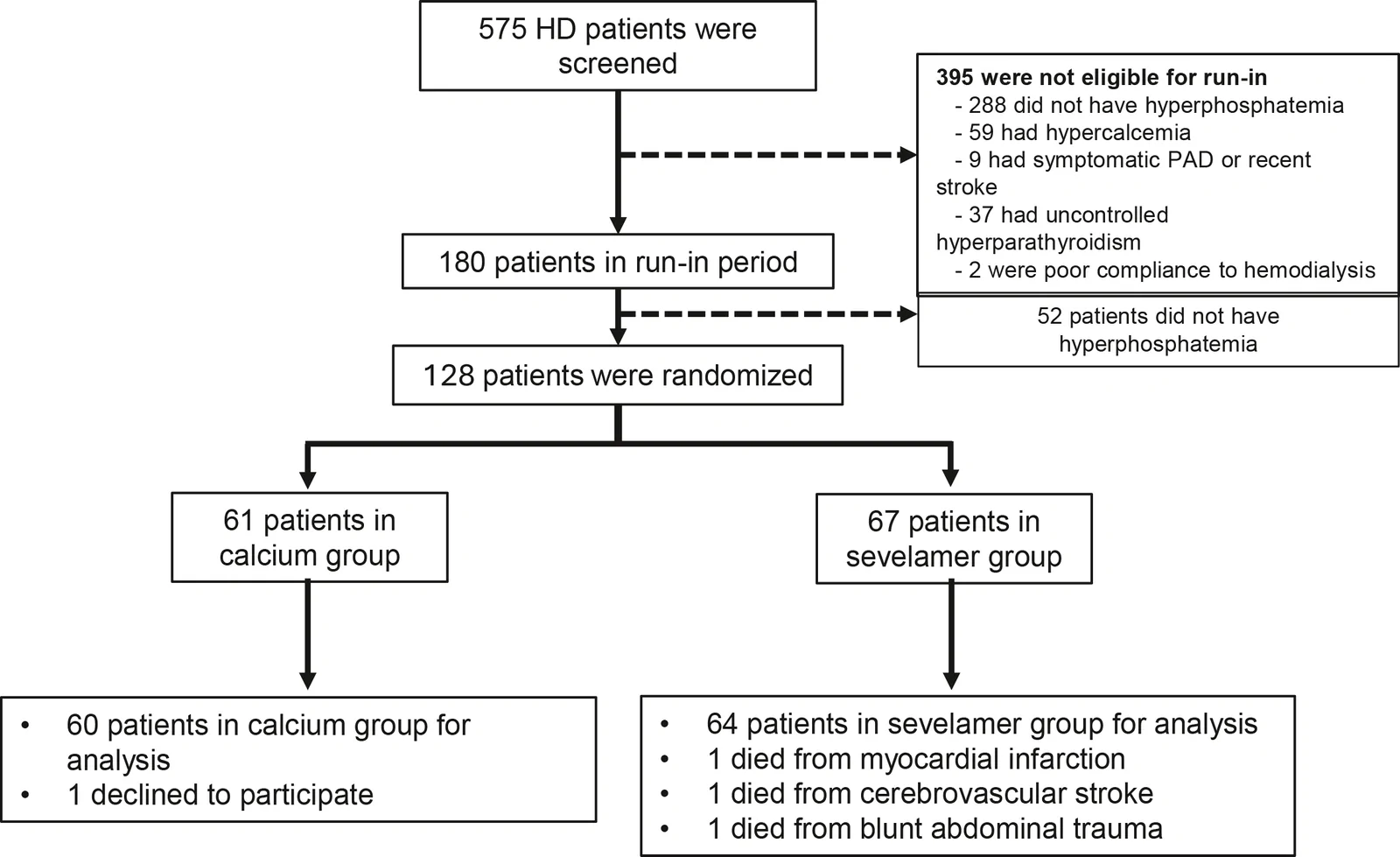
CONSORT diagram. Abbreviations: HD, hemodialysis; PAD, peripheral arterial disease.

The overall participants had a mean age of 53.0 ± 15.0 years, with 79 patients being male (61.7%). Forty-nine patients (38.3%) underwent HD twice weekly, whereas the remaining received thrice-a-week HD. Patients with RKF ≥100 mL/day comprised 47.7% of the population. Eighty-nine patients (72%) had a cardio-ankle vascular index greater than 8.0, indicating vascular stiffness, and 25 patients (19.5%) had asymptomatic peripheral vascular disease, with an ankle-brachial index of less than 0.9. The mean serum phosphate levels were 6.8 ± 1.7 mg/dL in the sevelamer group and 6.5 ± 1.1 mg/dL in the calcium group (Table 1). Baseline characteristics among participants with complete follow-up are provided in Table S1.

**Table 1:** Baseline Characteristics of Enrolled Participants

Baseline characteristics^a^	Calcium group (N = 61)	Sevelamer group (N = 67)	*P* Value
Age (y)	55.1 ± 14.5	51.2 ± 15.3	0.12
BMI (kg/m^2^)	22.2 (20.2-24.9)	22.8 (19.6-25.6)	0.83
Male sex	37 (60.7%)	42 (62.7%)	0.92
Dialysis vintage (y)	1.9 (0.8-3.8)	2.6 (0.9-5.1)	0.31
Cause of kidney failure			
Diabetic nephropathy	25 (41.0%)	21 (31.3%)	0.23
Hypertension	13 (21.3%)	13 (19.4%)	0.85
Glomerulonephritis	5 (8.2%)	14 (20.9%)	0.06
Unknown	15 (24.6%)	13 (19.4%)	0.53
Others	2 (3.3%)	5 (7.5%)	0.47
Comorbid condition			
Diabetes mellitus	27 (44.3%)	22 (32.8%)	0.11
Hypertension	57 (93.4%)	65 (97.0%)	0.36
Cerebrovascular disease	10 (16.4%)	9 (13.4%)	0.69
Coronary artery disease	7 (11.5%)	9 (13.4%)	0.89
History of heart failure	10 (16.4%)	18 (26.9%)	0.35
Current smoker	3 (4.9%)	0 (0.0%)	0.07
HD twice weekly	24 (39.3%)	25 (37.3%)	0.92
Single pool Kt/V	1.7 ± 0.4	1.7 ± 0.4	0.89
nPCR (g/kg/day)	1.3 ± 0.3	1.2 ± 0.2	0.19
Vascular access			0.33
Fistula	42 (68.9%)	51 (76.1%)	
Graft	1 (1.6%)	3 (4.5%)	
Tunneled catheter	18 (29.5%)	13 (19.4%)	
Baseline RKF ≥100 mL/day	29 (47.5%)	32 (47.8%)	0.88
Baseline RKF (ml/day)	50 (0, 800)	50 (0,300)	0.54
Midweek interdialytic weight gain (kg)	3.0 ± 1.4	2.8 ± 1.3	0.32
Patients complied to > 80% of medication (%)	48 (78.7%)	58 (86.6%)	0.09
Post parathyroidectomy	6 (9.8%)	9 (13.4%)	0.49
Previous phosphate binder			
Calcium carbonate	50 (82.0%)	47 (70.1%)	0.14
Sevelamer carbonate	2 (3.3%)	6 (9.0%)	0.92
Lanthanum	4 (6.6%)	4 (6.0%)	0.17
Aluminum hydroxide	5 (8.2%)	9 (13.4%)	0.31
Active vitamin D supplement	15 (24.6%)	20 (29.9%)	0.44
Ergocalciferol supplement	15 (24.6%)	11 (16.4%)	0.39
Calcimimetic agent	0 (0.0%)	4 (6.0%)	0.05
Total pCS (mg/L)	9.4 (5.7, 14.5)	7.6 (3.6, 13.8)	0.15
hs-CRP (mg/L)	2.7 (1.0, 7.7)	2.5 (0.8, 7.2)	0.76
iPTH (pg/mL)	422.9 (278.0, 409.7)	512.1 (242.9, 898.6)	0.69
Phosphate (mg/dL)	6.5 ± 1.1	6.8 ± 1.7	0.32
Corrected calcium (mg/dL)	8.8 ± 0.7	8.8 ± 0.8	0.76
Albumin (g/L)	3.9 ± 0.4	4.0 ± 0.4	0.41
HbA1c (%)	6.0 ± 1.4	6.1 ± 1.8	0.66
Total cholesterol (mg/dL)	156.8 ± 28.5	159.3 ± 40.4	0.70
LDL (mg/dL)	88.2 ± 28.7	92.4 ± 33.0	0.49
HDL (mg/dL)	44.9 ± 17.3	41.9 ± 14.0	0.36
CAVI	9.3 ± 1.7	8.8 ± 1.8	0.09
Cardio-ankle vascular index (CAVI) ≥ 9.0	34 (55.7%)	30 (44.8%)	0.14
Ankle-brachial index (ABI) < 0.9	11 (18.0%)	14 (20.9%)	0.35

Abbreviations: ABI, Ankle-brachial index (normal range, 0.9–1.2; <0.9 indicates peripheral arterial disease); BMI, body mass index; CAVI, cardio-ankle vascular index; ESKD, end-stage kidney disease; HbA1c, hemoglobin A1c; HD, hemodialysis; HDL, high-density lipoprotein; hs-CRP, high-sensitivity C-reactive protein; iPTH, intact parathyroid hormone; LDL, low-density lipoprotein; nPCR, normalized protein catabolic rate; pCS, p-cresyl sulfate; RKF, residual kidney function.

a Baseline characteristics are expressed as N (%), median (IQR) or mean ± SD.

### Changes in total p-cresyl sulfate concentration

The baseline total pCS levels were 7.6 mg/L (IQR 3.6, 13.8) in the sevelamer group and 9.4 mg/L (IQR 5.7, 14.5) in the calcium carbonate group (*P* = 0.15). At the 24-week primary endpoint, there was no significant difference in pCS change between the two groups (adjusted mean change 0.70 mg/L [95% CI –1.04 to 2.44] vs –0.09 mg/L [95% CI –1.70 to 1.51]; *P* = 0.07). Total pCS levels were not significantly changed in both groups over the 24-week period (Figure 2). Sensitivity analyses excluding 22 nonadherent patients (21.3% calcium; 13.4% sevelamer) yielded results consistent with the primary findings.

**Figure 2:**
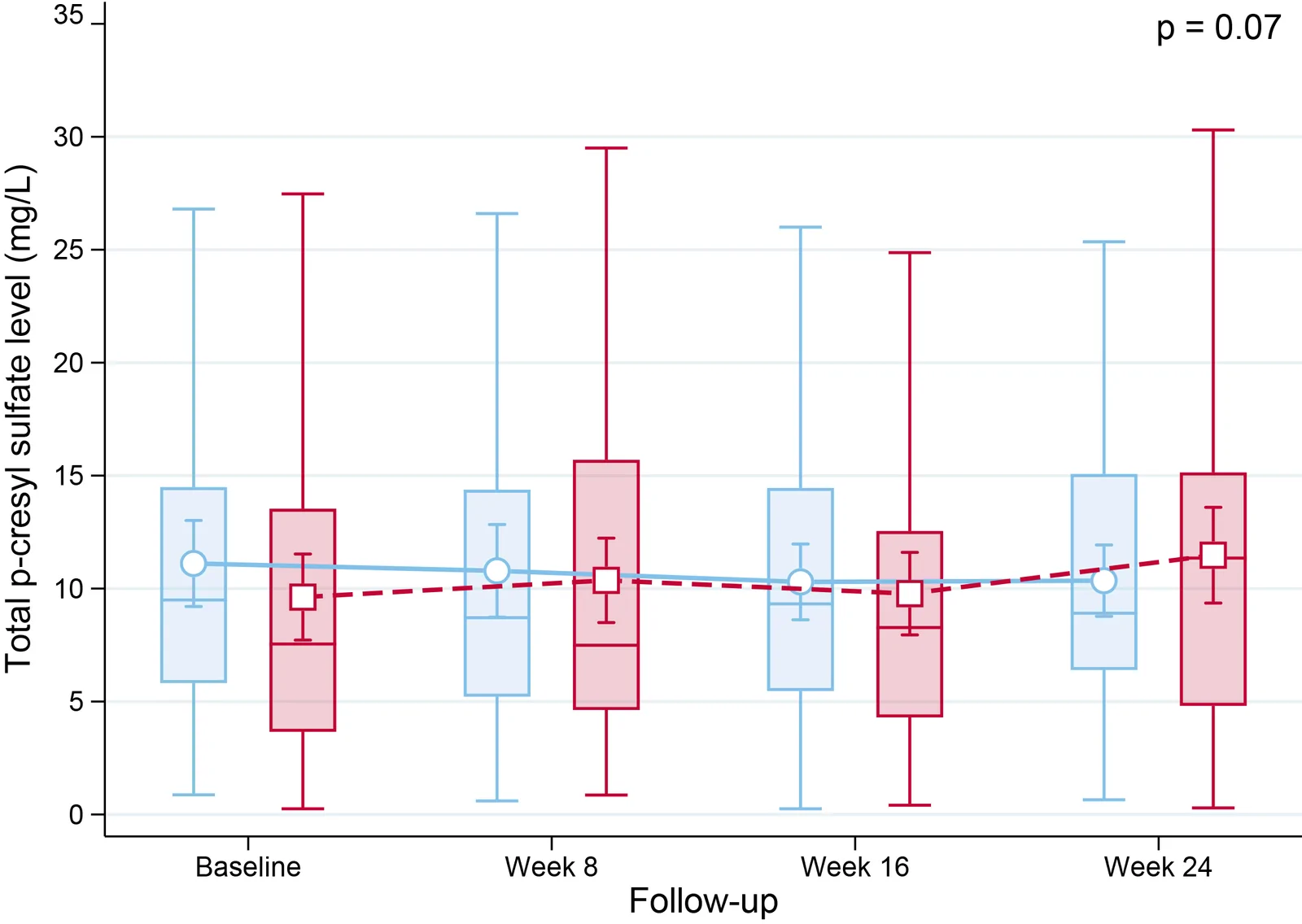
Changes in total p-cresyl sulfate levels over 24 weeks. Blue represents the calcium carbonate group and red represents the sevelamer group. Box plots show the median and interquartile range (IQR) of total p-cresyl sulphate levels. Lines and markers show adjusted geometric means derived by exponentiating least-squares means from the log-transformed model. *P* values were calculated using a linear mixed-effects model.

To explore potential treatment effect modifiers, subgroup analyses were performed. The pCS-reducing effect of sevelamer, compared with calcium, was more pronounced in patients with RKF ≥100 mL/day or those using high doses (sevelamer more than 4.8 g/day or calcium carbonate more than 3 g/day; *P* interaction = 0.04 and 0.002, respectively). No significant differences were observed based on phosphate levels or nPCR (Table S2).

In an exploratory multivariable logistic regression adjusting for phosphate binder type and dose, RKF, nPCR, and mean serum phosphate levels, several factors were independently associated with pCS changes. Preserved RKF and high-dose phosphate binders were independently associated with reduction of pCS over 24 weeks (adjusted OR 0.50 [95% CI 0.37-0.68], *P* < 0.001 and 0.50 [95% CI 0.35-0.71], *P* < 0.001, respectively). Conversely, patients with poor phosphate control (mean phosphate level > 5.5 mg/dL) were linked to increased pCS (adjusted OR 1.73 [95% CI 1.22-2.46], *P* = 0.002) (Table 2). Although subgroup analysis suggested a trend toward decreasing pCS in patients with RKF < 100 mL/day on high-dose sevelamer, this did not reach statistical significance (*P* = 0.27). The factors associated with reduced pCS are shown in Table S3.

**Table 2:** Factors Contributing to Increased Total p-Cresyl Sulfate Levels

Factors (N)^a^	Comparators (N)^a^	Univariate OR^b^ (95%CI)	*P* Value	Adjusted OR^b^^,^^c^ (95% CI)	*P* Value
Sevelamer (N = 64)	Calcium (N = 60)	1.22 (0.91, 1.62)	0.18	1.13 (0.84, 1.54)	0.39
RKF ≥ 100 mL/day (N = 60)	RKF<100 ml/day (N = 64)	0.49 (0.37, 0.66)	<0.001	0.50 (0.37, 0.68)	<0.001
Twice weekly HD (N = 47)	Thrice weekly HD (N = 77)	1.05 (0.78, 1.41)	0.74	-	-
nPCR > 1.2 g/kg/day (N = 62)	nPCR ≤ 1.2 g/kg/day (N = 62)	0.87 (0.63, 1.21)	0.24	0.81 (0.60, 1.09)	0.17
Mean serum phosphate > 5.5 mg/dL (N = 49)	Mean serum phosphate ≤ 5.5 mg/dL (N = 75)	1.22 (0.91, 1.64)	0.18	1.73 (1.22, 2.46)	0.002
High dose binders^d^ (N = 59)	Low dose binders^d^ (N = 65)	0.61 (0.46, 0.82)	0.001	0.50 (0.35, 0.71)	<0.001

Abbreviations: HD, hemodialysis; Mean P, mean phosphate level; nPCR, normalized protein catabolic rate; OR, odds ratio; RKF, residual kidney function.

a Sample sizes reflect the complete follow-up population (n=124). Four patients who dropped out are excluded.

b OR and adjusted OR below 1 means certain factor was associated with decreased p-cresyl sulfate levels, whereas OR > 1 mean certain factor was associated with increased p-cresyl sulfate levels.

c Adjusted ORs were reported from multivariable logistic regression adjusted for phosphate binder type (sevelamer vs calcium) and dose, RKF, nPCR, and mean serum phosphate levels.

d High dosages were predefined as mead dosage of sevelamer more than 4.8 g/day or calcium carbonate more than 3 g/day.

### Cardio-ankle vascular index change

Baseline cardio-ankle vascular index were 8.8 ± 1.8 in the sevelamer group and 9.2 ± 1.7 in the calcium group. After 24 weeks, there was slight increase in both groups (mean change 0.15 ± 0.16 in the sevelamer group versus 0.12 ± 0.17 in the calcium group). However, sevelamer was associated with attenuated increase of cardio-ankle vascular index than calcium group (adjusted mean change difference of –0.63 [95% CI –1.22 to –0.04], *P* = 0.04) after adjusting for baseline values (Table S4).

### Change in serum phosphate and calcium concentration

Serum phosphate reduction was similar between the two groups during each time point over 24 weeks (mean change –1.48 ± 1.70 mg/dL in the sevelamer group vs –1.39 ± 1.73 mg/dL in the calcium group, *P* value = 0.79). Interestingly, there was a statistically significant reduction in the overall mean serum calcium changes in sevelamer group over 24 weeks (adjusted mean change difference –0.14 [95% CI –0.26 to –0.03], *P* =0.01; Table S4).

### Change in serum hs-CRP levels

Baseline geometric means of hs-CRP level were 2.6 [95% CI 1.8–3.8] and 2.8 [95% CI 1.9-4.0] mg/L in the sevelamer and calcium carbonate groups, respectively. After 24 weeks follow-up, there were no significant difference hs-CRP level change between both groups (adjusted mean change difference –0.22 [95% CI –1.26 to 0.82], *P* = 0.36; Table S4).

### Biochemical changes

Over the 24-week period, the CKD-MBD parameters (specifically intact PTH and alkaline phosphatase levels), liver enzyme, hemoglobin, and serum albumin levels remained stable. However, we observed a significant reduction in total cholesterol level in the sevelamer group compared with the calcium group (adjusted mean difference –8.50 mg/dL [95% CI –16.80, -0.21], *P* = 0.05). A minor but statistically significant increase in serum albumin change was observed with sevelamer (adjusted mean difference 0.06 g/dL [95% CI 0.01-0.13], *P* = 0.02) Detailed results are available in Table S4.

### Adverse events

Among the 128 patients, 3 deaths occurred in the sevelamer group (one cardiovascular, one cerebrovascular, one traumatic), none attributed to study treatment. Minor adverse events were comparable between the two groups. Constipation was reported by 12 patients (17.9%) in the sevelamer group and 13 patients (19.7%) in the calcium group (*P* = 0.63). Only 1 patient in the calcium group reported nausea (Table S5).

## Discussion

In this study, we investigated the effects of sevelamer on pCS levels, as well as cardio-ankle vascular index in HD patients with hyperphosphatemia. The aims were to compare the pCS lowering and pleiotropic effects of sevelamer, a noncalcium-based phosphate binder, to calcium carbonate. After 24 weeks of sevelamer treatment, pCS levels were not statistically different from calcium carbonate treatment. However, it attenuated cardio-ankle vascular index progression and reduced total cholesterol levels, suggesting pleiotropic vascular benefit independent of PBUT lowering.

Several studies have shown that sevelamer is effective in lowering pCS in predialysis CKD patients when combined with dietary protein restriction.^17^^,^^24^ In such study, all patients adhered to a low protein intake of 0.68-0.72 g/kg/day. Conversely, the RCT by Bennis et al^23^ did not find the effectiveness of sevelamer in reducing pCS in predialysis CKD, but their patients had significantly lower baseline pCS levels than other studies. Patients receiving long-term HD face a uniquely unfavorable pCS balance: higher protein intake requirements, progressive RKF loss, which contributes up to 50% of pCS excretion, and dialysis-related gut microbiome disruption all drive toxin accumulation.^5^^,^^6^^,^^22^^,^^27^, ^28^, ^29^, ^30^ Furthermore, dialysis-related factors, such as inflammation, oxidative stress, and electrolyte and fluid disturbances, can lead to increased constipation and eventually gut permeability to pCS.^29^ Such various factors are contributed to higher pCS levels in long-term HD than pre-dialysis CKD patients, making evidence from pre-dialysis trials not directly applicable to the HD setting.^31^, ^32^, ^33^ The clinical significance of pCS reduction remains incompletely defined. A recent study demonstrated that each 1 mg/L increase in pCS was independently associated with peripheral arterial stiffness (adjusted odds ratio 1.24, 95% CI 1.12-1.37).^34^ In our study, the adjusted mean change in pCS was less than 1 mg/L across both groups; consequently, this magnitude of change may be insufficient to produce measurable vascular effects.

Significant phosphate reduction was observed in both groups after the study drugs were initiated, reflecting good patient adherence. Serum calcium levels were slightly higher in the calcium carbonate group. Because hypercalcemia is one of the risk factors for vascular calcification and all-cause and cardiovascular mortality in HD patients, thus the effect calcium reduction is beneficial in those having hypercalcemia that can further reduce vascular calcification and all-cause mortality.^35^, ^36^, ^37^, ^38^, ^39^

Our study did not find a pCS-reducing effect of sevelamer in HD setting. However, the cohort study by Lin et al^18^ of patients receiving long-term HD reported conflicting results, demonstrating that sevelamer effectively reduced pCS levels over 12 weeks. This discrepancy may be due to the longer dialysis vintage in the study by Lin et al^18^, which had a mean duration of 5.3 ± 1.1 years. Although the baseline RKF was not reported, patients with a longer dialysis vintage are usually anuric.^20^ The baseline pCS levels in the study by Lin et al^18^ were also approximately 4 times higher than in our study, with a mean of 31.3 ± 10.6 mg/L. This finding supports the idea that RKF is crucial for PBUT elimination and that HD is ineffective at removing them, leading to gradual accumulation of pCS over time. Apart from the impact of RKF, the dosage of sevelamer may affect pCS reduction in a dose-dependent manner. Our study found that RKF and high doses of sevelamer had a reducing effect on pCS levels. We also analyzed the effects of pCS reduction based on combined-factor subgroup analysis using RKF and phosphate binder dosages. Sevelamer showed a trend of pCS lowering in patients with RKF <100 mL/day and using high dosages of the medication. However, this was not statistically significant, likely because of the limited sample size. In such situation, in which native kidney excretion is significantly depleted, intestinal adsorbents may be crucial strategies for reducing PBUTs.

The cardio-ankle vascular index serves as a robust marker of arterial stiffness, influenced by various age, comorbid conditions, blood pressure, visceral fat area, and CKD-MBD-related vascular calcification.^40^, ^41^, ^42^, ^43^ Higher cardio-ankle vascular index correlates with cardiac diastolic dysfunction and a 20% to 50% increase in cardiovascular events per 1 unit increase.^41^^,^^42^^,^^44^^,^^45^ To minimize hemodialysis-related variability, cardio-ankle vascular index was measured before the mid-week HD sessions; interdialytic weight gain remained similar between groups. Although both groups showed longitudinal increases in the cardio-ankle vascular index—consistent with the failure to reduce pCS—the increase was significantly, albeit modestly, attenuated in the sevelamer group. These findings suggest that sevelamer may influence arterial stiffness through pleiotropic mechanisms independent of pCS reduction. Two mechanisms likely contribute. The first mechanism involves calcium avoidance. The calcium group experienced significantly higher serum calcium level increases, a well-established driver of vascular calcification, supported by prior meta-analyses showing lower coronary calcification with noncalcium binders.^46^ Second, the lipid-lowering effect of sevelamer is evidenced by significant total cholesterol reduction in our study, which may further attenuate arterial stiffness progression through improved endothelial function.^47^

Although sevelamer has been shown to sequester gut-derived endotoxins and lower hs-CRP in vitro, our study found no significant difference between groups.^48^, ^49^, ^50^ This contrasts with Stinghen et al^49^, who reported a marked hs-CRP reduction after switching from calcium carbonate to sevelamer in HD patients with a baseline hs-CRP of 4.8 ± 1.2 mg/L — nearly double our cohort’s baseline level. This discrepancy likely reflects a floor effect, in which lower baseline inflammation limits the detectable anti-inflammatory benefit. Additionally, their patients had fewer diabetics (10%) and a shorter dialysis vintage. Inflammation in patients receiving HD can be influenced by factors like comorbid conditions, dialysis vintage, catheter use, and dialyzer issues.^51^ In our study, 24% of patients had tunneled dialysis catheters, possibly confounding the hs-CRP levels.

Three deaths occurred in the sevelamer group, including one cardiovascular, one cerebrovascular, and one traumatic. None of these were considered related to study treatment given the patients’ pre-existing comorbid conditions. Minor adverse events were similar between groups. Constipation was the most frequently reported, occurring in 17.9% and 19.7% of the sevelamer and calcium groups, respectively (*P* = 0.63). These findings suggest a comparable short-term safety profile, though long-term safety data for sevelamer in HD patients remain limited.

### Limitations

Several limitations of this study warrant consideration. First, pCS is a surrogate marker for cardiovascular events rather than a hard clinical endpoint. Second, the observed pCS change did not reach the clinically significant threshold of 1 mg/L, suggesting that the study may have been underpowered or that the sevelamer dose and treatment duration were insufficient. Third, although a trend toward pCS reduction was noted in patients with RKF < 100 mL/day and those receiving high-dose sevelamer, the sample size was inadequate to confirm this exploratory finding.

In conclusion, in HD patients receiving phosphate binders titrated to control serum phosphate, pCS levels did not change significantly after 24 weeks with either sevelamer or calcium carbonate. However, the increase in cardio-ankle vascular index was smaller with sevelamer, independent of pCS change. Preserved RKF and high-dose phosphate binders were associated with pCS reduction, whereas poor phosphate control was associated with increased pCS levels. A trend toward greater pCS reduction with sevelamer was observed in patients with RKF <100 mL/day and those receiving high-dose phosphate binders. Further studies are needed to evaluate high-dose sevelamer in patients with loss of residual kidney function.
