# Supplementary material for: Protein-bound Uremic Toxin Lowering Effects of Sevelamer in Hyperphosphatemic Hemodialysis Patients: A Multicenter Randomized Controlled Trial
Source: Kidney Med. 2026 Sep 11;8(11):101527. doi: 10.1016/j.xkme.2026.101527 (PMC13717100; doi:10.1016/j.xkme.2026.101527)
Supplement: Supplementary file 1 [file mmc1.pdf]

Online Supplementary Materials

for

Protein-bound uremic toxin lowering effects of sevelamer in hyperphosphatemic hemodialysis patients: a multi-center randomized controlled trial

By

Napas Srifa, Jeerath Phannajit, Patchara Tanateerapong, Kamonrat Chongthanakorn, Sipanan Thamratnopkoon, Wannasit Wathanavasin, Supat Vanichakarn, Sookruethai Lekhyananda, Yingyos Avihingsanon, Kearkiat Praditpornsilpa, Somchai Eiam-Ong, and Paweena Susantitaphong

Appendix S1: Protocol-based phosphate binder dose titration protocol

Sevelamer carbonate (Kavolred®) 800 mg and calcium carbonate (Chalkcap®) 1,000 mg (containing 400 mg elemental calcium) were used in this study. Doses were titrated according to serum phosphate levels, with a target serum phosphate range of 3.5-5.5 mg/dL.

Initiation period

For serum phosphate levels of 5.6-6.5 mg/dL, patients received sevelamer 800 mg three times daily or calcium carbonate 500 mg three times daily with meals. For levels of 6.6-7.5 mg/dL, the doses were increased to sevelamer 1,600 mg three times daily or calcium carbonate 1,000 mg three times daily. For serum phosphate levels >7.5 mg/dL, patients received sevelamer 2,400 mg three times daily or calcium carbonate 1,500 mg three times daily.

Follow-up period

At follow-up visits, serum phosphate levels and pill counts were assessed. If serum phosphate was <3.5 mg/dL, the dose was reduced by one step (sevelamer 800 mg three times daily or calcium carbonate 500 mg three times daily). If serum phosphate was 3.5-5.5 mg/dL, the same dose was maintained. If serum phosphate remained >5.5 mg/dL, the dose was increased by one step, up to a maximum of sevelamer 3,200 mg three times daily or calcium carbonate 2,000 mg three times daily. For persistent hypophosphatemia (<2.5 mg/dL), the dose was reduced by 50%.

Serum calcium was also monitored, and dialysate calcium was adjusted to maintain serum calcium levels between 8.3 and 10.5 mg/dL. In patients receiving calcium carbonate, the dose was reduced by 50% if hypercalcemia (≥ 10.5 mg/dL) occurred. Aluminum hydroxide was used as rescue therapy if serum phosphate remained ≥ 5.5 mg/dL.

Table S1: Baseline characteristics only completed follow-up participants

Baseline characteristics	Calcium carbonate group (N=60)	Sevelamer group (N=64)
**Age (years)**	54.7 ± 14.3	50.5 ± 15.2
**BMI (kg/m2)**	22.2 (20.2-15.2)	22.8 (19.6-25.4)
**Male sex**	37 (61.7%)	40 (62.5%)
**Dialysis vintage (years)**	1.9 (0.9-4.1)	2.7 (0.9-5.2)
**Cause of ESKD**		
- Diabetic nephropathy	24 (40.0%)	19 (29.7%)
- Hypertension	13 (21.7%)	13 (20.3%)
- Glomerulonephritis	5 (8.3%)	13 (20.3%)
- Unknown	15 (25.0%)	13 (20.3%)
- Others	2 (3.3%)	5 (7.8%)
**Comorbidities**		
- Diabetes mellitus	26 (43.3%)	19 (29.7%)
- Hypertension	56 (93.3%)	62 (96.9%)
- Cerebrovascular disease	10 (16.7%)	9 (14.1%)
- Coronary artery disease	7 (11.7%)	8 (12.5%)
- History of heart failure	10 (16.7%)	15 (23.4%)
- Current smoker	3 (5.0%)	0 (0.0%)
**HD twice weekly**	23 (38.3%)	24 (37.5%)
**Single pool Kt/V**	1.7 ± 0.4	1.7 ± 0.4
**nPCR (g/kg/day)**	1.3 ± 0.3	1.2 ± 0.2
Vascular access		
- Fistula	41 (68.3%)	48 (75.0%)
- Graft	1 (1.7%)	3 (4.7%)
- Tunneled catheter	18 (30.0%)	13 (20.3%)
**Baseline RKF ≥100 ml/day**	28 (46.7%)	29 (45.3%)
**Post parathyroidectomy**	6 (10.0%)	9 (14.1%)
**Previous phosphate binder**		
- calcium carbonate	49 (81.7%)	46 (71.9%)
- sevelamer carbonate	2 (3.3%)	5 (7.8%)
- lanthanum	4 (6.7%)	4 (6.2%)
- aluminium hydroxide	5 (8.3%)	9 (14.1%)
**Active vitamin D supplement**	15 (25.0%)	20 (31.2%)
**Ergocalciferol supplement**	14 (23.3%)	11 (17.2%)
**Calcimimetic agent**	0 (0.0%)	4 (6.2%)
**Baseline total pCS (mg/L)**	9.5 (5.8, 14.5)	7.5 (3.7, 13.5)
**Baseline hs-CRP (mg/L)**	2.6 (1.0, 8.0)	2.2 (0.8, 7.2)
**Baseline iPTH (pg/mL)**	424.5 (279.8, 733.1)	512.8 (251.9, 886.5)
**Baseline PO4 (mg/dL)**	6.5 ± 1.1	6.8 ± 1.7
**Baseline corrected Ca (mg/dL)**	8.8 ± 0.7	8.8 ± 0.8
**Baseline albumin (g/L)**	3.9 ± 0.4	4.0 ± 0.4
**Baseline HbA1c (%)**	5.9 ± 1.4	5.8 ± 1.4
**Baseline LDL-C (mg/dL)**	87.9 ± 28.8	91.7 ± 32.3
**Baseline HDL-C (mg/dL)**	45.1 ± 17.3	42.5 ± 14.0
**Baseline CAVI**	9.2 ± 1.7	8.8 ± 1.8
**Baseline ABI < 0.9**	11 (18.3%)	13 (20.3%)

Baseline characteristics are expressed as N (%), median (IQR) or mean ± SD
Sample sizes reflect the complete follow-up population (n=124). Four patients who dropped out are excluded.
Abbreviations and definitions: BMI; body mass index, ESKD; end-stage kidney disease, HD; hemodialysis, nPCR; normalized protein catabolic rate, RKF; residual kidney function, pCS; p-cresyl sulfate, hs-CRP; high sensitivity C-reactive protein, iPTH; intact parathyroid hormone, HbA1c; glycated hemoglobin, LDL; low density lipoprotein, HDL; high density lipoprotein, CAVI; cardio-ankle cascular index, ABI; ankle-brachial index (normal range 0.9-1.2, <0.9 indicating peripheral arterial disease)

Table S2: Weekly geometric mean ratio (GMR) of total p-cresyl sulfate in complete follow-up population (N=124)

Model analysis/ subgroups	Weekly gemotric mean ratio of total p-cresyl sulphate (95%CI)		P interaction
	Calcium group	Sevelamer group	
**Subgroup by RKF**			
**RKF < 100 ml/day (N=64)**	1.01 (0.99, 1.02)	1.02 (1.01 1.04)*	**0.04**
**RKF ≥ 100 ml/day (N=60)**	0.99 (0.98, 1.00)	0.99 (0.98, 1.01)	
**Subgroup by mean phosphate levels**			
**Mean phosphate level ≤ 5.5 mg/dL (N=75)**	0.99 (0.98, 1.01)	1.01 (1.00, 1.02)	0.60
**Mean phosphate level > 5.5 mg/dL (N=49)**	1.00 (0.99, 1.02)	1.00 (0.99, 1.02)	
**Subgroup by phosphate binder doses**			
**High dose phosphate binders (N=59)**	1.00 (0.99, 1.01)	0.99 (0.98, 1.00)	**0.002**
**Low dose phosphate binders (N=65)**	1.00 (0.99, 1.01)	1.02 (1.01, 1.04)*	
**Subgroup by nPCR**			
**nPCR > 1.2 g/kg/day (N=59)**	0.99 (0.98, 1.01)	1.01 (1.00, 1.02)	0.51
**nPCR ≤ 1.2 g/kg/day (N=65)**	1.00 (0.99, 1.01)	1.01 (1.00, 1.02)	

Geometric mean ratio (GMR) > 1 means increased, < 1 means decreased and =1 means unchanged. *significantly changed ($p < 0.05$)
Sample sizes reflect the complete follow-up population (n=124). Four patients who dropped out are excluded.
Abbreviations and definitions: RKF; residual kidney function, pCS; p-cresyl sulfate, nPCR; normalized protein catabolic rate
High dose phosphate binders means daily sevelamer carbonate more than 4,800 mg or calcium carbonate more than 3,000 mg

Table S3: Subgroup analyses of univariate and multivariable logistic regression comparing effects of sevelamer with calcium carbonate

Subgroups	Univariate OR [b] (95%CI)	p-value	Adjusted OR [b,c] (95%CI)	p-value
RKF < 100 ml/day and High dose [d] phosphate binders (N=28) [a]	0.40 (0.08, 1.90)	0.25	0.41 (0.08, 2.04)	0.27
RKF < 100 ml/day and Low dose [d] phosphate binders (N=36) [a]	1.48 (0.35, 6.25)	0.59	1.64 (0.35, 7.64)	0.53
RKF ≥ 100 ml/day and High dose [d] phosphate binders (N=31) [a]	1.17 (0.28, 4.78)	0.83	1.21 (0.28, 5.21)	0.80
RKF ≥ 100 ml/day and Low dose [d] phosphate binders (N=29) [a]	2.06 (0.46, 9.13)	0.34	2.30 (0.48, 11.05)	0.30

[a] Sample sizes reflect the complete follow-up population (n=124). Four patients who dropped out are excluded.

[b] Odds ratio (OR) and adjusted OR comparing Sevelamer vs Calcium carbonate below 1 means certain factor was associated with decreased p-cresyl sulfate while OR > 1 mean certain factor was associated with increased p-cresyl sulfate.

[c] Adjusted ORs were reported from multivariable logistic regression adjusted for phosphate binder type (sevelamer vs calcium) and dose, RKF, nPCR, and mean serum phosphate.

[d] high dosages were predefined as sevelamer more than 4.8 g/day or calcium carbonate more than 3 g/day

Abbreviations: RKF; residual kidney function, nPCR; normalized protein catabolic rate, HD; hemodialysis, Mean P; mean phosphate level, high dose binders; mean sevelamer dose > 4.8 g/day or calcium carbonate dose > 3 g/day

Table S4: Biochemistry and CAVI changes between groups over 24 weeks in the complete follow-up population

Parameters	Calcium groups (N=60)		Sevelamer group (N=64)		Adjusted mean change difference (95%CI)	P-value between groups
	Baseline	Week 24	Baseline	Week 24		
**CKD-MBD parameters and liver enzymes**						
**Phosphate (mg/dL)**	6.5 ± 1.1	5.1 ± 1.4	6.8 ± 1.7	5.2 ± 1.7	0.04 (-0.24, 0.31)	0.79
**Calcium (mg/dL)**	8.8 ± 0.7	**9.0 ± 0.8***	8.8 ± 0.8	**8.8 ± 0.8***	**-0.14 (-0.26, -0.03)**	**0.01**
**iPTH (pg/mL)**	419.3 (333.8,562.7)	398.5 (304.8, 520.9)	376.2 (269.1, 525.8)	418.3 (287.4, 609.0)	68.43 (-44.75, 181.61)	0.84
**ALP (IU/L)**	108.6 ± 81.8	141.4 ± 169.8	110.4 ± 69.7	160.3 ± 113.5	10.17 (-18.99, 39.32)	0.49
**ALT (IU/L)**	13.0 ± 7.5	13.7 ± 8.1	14.0 ± 9.8	15.1 ± 13.8	1.18 (-1.36, 3.72)	0.36
**AST (IU/L)**	17.5 ± 8.5	20.1 ± 8.3	17.5 ± 8.1	22.5 ± 18.1	1.14 (-1.81, 4.10)	0.45
**Inflammation and anemia**						
**hs-CRP (mg/L)**	2.8 (1.9, 4.0)	2.6 (1.8, 3.6)	2.6 (1.8, 3.8)	2.3 (1.6, 3.5)	-0.22 (-1.26, 0.82)	0.36
**Albumin (g/dL)**	3.9 ± 0.4	**3.8 ± 0.3***	4.0 ± 0.4	**3.9 ± 0.4***	**0.06 (0.01, 0.13)**	**0.02**
**Hemoglobin (g/dL)**	9.6 ± 1.3	9.8 ± 1.4	9.7 ± 1.6	9.6 ± 1.4	-0.06 (-0.41, 0.31)	0.76
**Lipid profiles**						
**Cholesterol (mg/dL)**	156.7 ± 28.7	**149.9 ± 26.6***	159.1 ± 40.2	**130.4 ± 27.9***	**-8.50 (-16.80, -0.21)**	**0.05**
**LDL(mg/dL)**	87.9 ± 28.8	85.1 ± 26.7	91.7 ± 32.3	68.2 ± 26.1	-6.47 (-13.93, 0.99)	0.09
**HDL (mg/dL)**	45.1 ± 17.3	45.0 ± 13.7	42.5 ± 14.0	44.5 ± 14.3	-1.52 (-5.23, 2.19)	0.42
**Vascular stiffness**						
**Cardio-ankle cascular index (CAVI)**	9.2 ± 1.7	**9.5 ± 1.4***	8.8 ± 1.8	**9.0 ± 1.6***	**-0.63 (-1.22, -0.04)**	**0.04**

Baseline and 24th-week data are expressed in mean ± SD or geometric mean (95%CI).
Sample sizes reflect the complete follow-up population (n=124). Four patients who dropped out are excluded.
Mean change difference is adjusted for baseline values using ANCOVA.
Abbreviations: CKD-MBD; chronic kidney diseae bone mineral disorders, iPTH; intact parathyroid hormone, ALP; alkaline phosphatase, ALT; alanine transaminase, AST; aspartate transaminase, hs-CRP; high sensitivity C-reactive protein, LDL; low density lipoprotein, HDL; high density lipoprotein.

Table S5: Adverse effects

Adverse effects	Calcium group (N=61)	Sevelamer group (N=67)	P-value
**Death**	0 (0.0%)	3 (4.5%)†	0.10
**Constipation**	12 (19.7%)	12 (17.9%)	0.63
**Dizziness**	0 (0.0%)	0 (0.0%)	-
**Nauseous**	1 (1.6%)	0 (0.0%)	0.29
**Diarrhea**	0 (0.0%)	0 (0.0%)	-

Data are expressed are expressed as N (%)

†one patient died from myocardial infarction at 2 weeks, another from stroke at 9 weeks, and the third from blunt abdominal trauma at 8 weeks follow-up
